# Development of Ni-doped A-site lanthanides-based perovskite-type oxide catalysts for CO_2_ methanation by auto-combustion method

**DOI:** 10.1039/d4ra02106a

**Published:** 2024-06-25

**Authors:** Muddasar Safdar, Nasir Shezad, Farid Akhtar, Harvey Arellano-García

**Affiliations:** a Department of Process and Plant Technology, Brandenburg University of Technology (BTU) Cottbus-Senftenberg Platz der Deutschen 1 03046 Cottbus Germany arellano@b-tu.de; b Department of Chemical Engineering Technology, Government College University Faisalabad: GCUF Allama Iqbal Road Faisalabad Punjab 38000 Pakistan; c Department of Engineering Science and Mathematics, Division of Materials Science, Luleå University of Technology 97187 Luleå Sweden farid.akhtar@ltu.se

## Abstract

Engineering the interfacial interaction between the active metal element and support material is a promising strategy for improving the performance of catalysts toward CO_2_ methanation. Herein, the Ni-doped rare-earth metal-based A-site substituted perovskite-type oxide catalysts (Ni/AMnO_3_; A = Sm, La, Nd, Ce, Pr) were synthesized by auto-combustion method, thoroughly characterized, and evaluated for CO_2_ methanation reaction. The XRD analysis confirmed the perovskite structure and the formation of nano-size particles with crystallite sizes ranging from 18 to 47 nm. The Ni/CeMnO_3_ catalyst exhibited a higher CO_2_ conversion rate of 6.6 × 10^−5^ mol_CO_2__ g_cat_^−1^ s^−1^ and high selectivity towards CH_4_ formation due to the surface composition of the active sites and capability to activate CO_2_ molecules under redox property adopted associative and dissociative mechanisms. The higher activity of the catalyst could be attributed to the strong metal–support interface, available active sites, surface basicity, and higher surface area. XRD analysis of spent catalysts showed enlarged crystallite size, indicating particle aggregation during the reaction; nevertheless, the cerium-containing catalyst displayed the least increase, demonstrating resilience, structural stability, and potential for CO_2_ methanation reaction.

## Abstract 2

Improving the performance of perovskite-supported Ni catalysts for CO_2_ conversion into methane by A-site doping of lanthanides.

## Graphical abstract

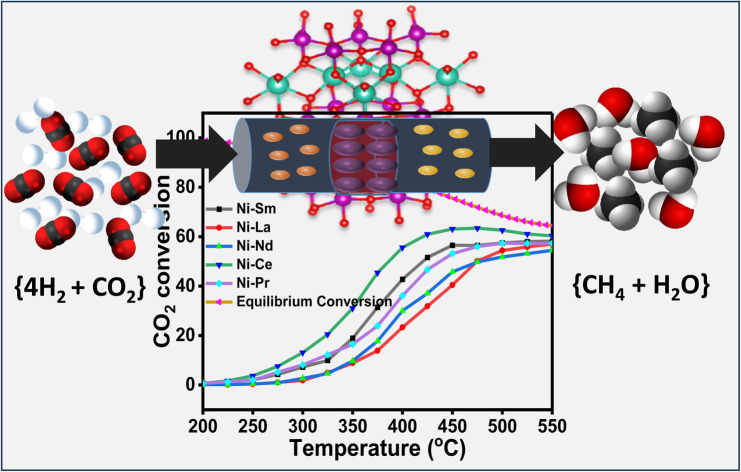

## 1. Introduction

Fossil fuel utilization to fulfill domestic and industrial energy demands has triggered the emission of greenhouse gases, mainly carbon dioxide (CO_2_). The CO_2_ emissions have aggravated global warming, badly affecting life on Earth. The scientific community is committed to addressing the global warming problem by reutilizing atmospheric CO_2_ through carbon capture and utilization (CCU) as well as carbon capture and storage (CCS) technologies.^1,2^ Among the adopted routes, catalytic CO_2_ conversion has received significant attention to produce renewable fuels and commodity chemicals.^3^ In this technique, the Power-to-Gas (P2G) is the most suitable way for the hydrogenation and production of synthetic natural gas (SNG) *via* the so-called Sabatier reaction (eqn (1)).^4–6^

1 CO_2_ + 4H_2_ ↔ CH_4_ + 2H_2_O, Δ*H*_298 K_ = −164.7 kJ mol^−1^

CO_2_ hydrogenation is thermodynamically an exothermic reaction favored under mild conditions like low reaction temperature and atmospheric pressure.^7^ In addition, high temperature affects the selectivity of the product. It increases the carbon monoxide (CO) production through various side reactions *i.e.* reverse water gas shift reaction (RWGS) (CO_2_ + H_2_ ↔ CO + H_2_O), Reverse-Boudouard (RB) reaction (C + CO_2_ ↔ 2CO). The RB and other side reactions can result in the deactivation of the catalyst due to carbon deposition. Therefore, an active and suitable catalytic system is required to achieve high conversion, selectivity, stability, and appropriate reaction rate.^8–10^

According to the literature, Ni-supported catalysts have extraordinary merits due to their lower cost, higher selectivity, and better stability. Their catalytic properties can further be improved by incorporating redox support materials like perovskite-type oxides (PTOs) to tune the oxygen vacancies, resilience against coke formation, metal–support interaction, and metal dispersion.^11–13^ The Ni-based PTOs are emerging materials with the general formula ABO_3_ (where A cations are alkaline-earth, rare-earth, and B sites are composed of transition metals) and unique characteristics for CO_2_ methanation.^14–16^ Regarding Ni-based PTOs, studies have reported that the rare earth elements (La, Ce, Sm, *etc.*) enhance the CO_2_ activation and act as a modifier to tune the basicity, metal dispersion, and metal–support interaction. Adding La_2_O_3_ intensifies the CO_2_ chemisorption, resulting in H_2_ dissociation and CO_2_ activation at a lower temperature.^17^ Similarly, cerium oxide (CeO_2_), samarium oxide (Sm_2_O_3_), and praseodymium oxide (Pr_*n*_O_2*n*−*n*_) have been employed as catalytic promotors to tune the metal–support interaction. Further, promotors enhance the surface basicity due to the interaction of their oxygen vacancies with the oxygen atom of the CO_2_ molecule, weakening the C=O bond strength^18^ and, subsequently, enhancing the catalytic activity of Ni-based systems towards CO_2_ hydrogenation. Likewise, it has been reported that manganese (Mn) substitution at the B sites enhances the oxygen exchange capacities within the PTOs structures, favoring the optimal catalytic performance as reported for Ni/YMn_1−*x*_Al_*x*_O_3_ perovskite catalysts.^6^ Perovskites ABO_3_ with substituted Mn species at B sites might create the defect chemistry within PTOs structure, thereby promoting the number of non-stoichiometric vacancies and enhancing the lattice oxygen.^19^ Keeping the fixed B position, variation at the A site of perovskite material leads to improved surface oxygen concentration, specific surface area and lowers the reduction temperature of the catalysts.^20^ To our knowledge, introducing lanthanide metals at A-sites while keeping Mn at B-sites in PTOs has rarely been explored for CO_2_ hydrogenation. In view of the above, the present work reports different perovskite-type supports (A_*x*_Mn_*y*_O_3_, A = Sm, La, Nd, Ce, Pr with *x*, *y* = 1) prepared by A-site substitution of rare earth metals through one-pot auto-combustion method followed by Ni loading *via* impregnation method. The prepared catalysts were investigated for hydrogenation of CO_2_ into CH_4_. Thus, the behavior and potential of 10% by wt Ni/AMnO_3_ perovskites series of catalysts were evaluated. The higher performance exhibited by the Ni/CeMnO_3_ catalyst showed the potential of PTOs for CO_2_ methanation.

## 2. Experimental section

### 2.1 Catalyst synthesis

Analytical-grade samarium(iii) nitrate hexahydrate, lanthanum(iii) nitrate hexahydrate, neodymium(iii) nitrate hexahydrate, and praseodymium(iii) nitrate hexahydrate were purchased from Sigma Aldrich, while cerium(iii) nitrate hexahydrate was purchased from Merck. Manganese nitrate hexahydrate and nickel nitrate hexahydrate were purchased from Alfa Aesar.

Perovskite oxides A_*x*_Mn_*y*_O_3_ (*x*, *y* = 1, rare earth elements denoted as A, A = Sm, La, Nd, Ce, Pr) were prepared by the one-pot auto-combustion method. Initially, the stoichiometric amounts of each rare earth metals (A = Sm, La, Nd, Ce, Pr) were mixed with Mn(NO_3_)_2_·4H_2_O separately as a precursor and dissolved in deionized water and stirred for 60 min to obtain a homogeneous mixture. Then, the citric acid (CA, VWR) as a fuel was dissolved with a molar ratio CA : Mn : A = 2 : 1 : 1 and stirred for 30 min, followed by the addition of ethylene glycol (EG, Merck) to the above mixture with a molar ratio of EG : CA = 1.2 : 1 and stirred for 2 h at 100 °C. Subsequently, the solution temperature was gradually increased to 350 °C with a fixed time interval (20 min) to evaporate the solvents until a gel-like material was obtained. Upon further heating, the gel was transformed into a foam-like structure, which self-ignited and turned into flakes. The flakes were further crushed to raw powder, dried at 120 °C, and calcined in air at 900 °C for 4 h with a heating rate of 10 °C min^−1^.^21^ The obtained perovskite-like supports SmMnO_3_, LaMnO_3_, NdMnO_3_, CeMnO_3_, and PrMnO_3_ were referred to as SM, LM, NM, CM, and PM.

To obtain Ni-doped PTOs, 10% by wt Ni was impregnated over the prepared support using the wetness impregnation method.^16^ The Ni precursor dissolved in de-ionized water was added to the perovskite supports and vigorously stirred on a hot plate at 100 °C until the solvent was evaporated entirely. The obtained materials were dried overnight at 120 °C, calcined at 500 °C (10 °C min^−1^) for 4 h in air, ground, and sieved accordingly. The synthesized catalysts were named NSM, NLM, NNM, NCM, and NPM, respectively.

### 2.2 Characterization

The chemical composition of the samples was measured by ICP-AES and ICP-SFMS. The samples were digested in nitric acid and analyzed in an Agilent 4200 MP-AES for the measurements. The crystallinity of the PTOs was analyzed by X-ray diffraction using the Cu-based anode (Cu-Kα radiation, *λ* = 1.5418 Å) with a Bruker D2 Phaser Instrument. The diffractograms were acquired in the 2*θ* range of 10 to 80° with step size of 0.02° and step time of 0.35 s, respectively. The crystallite sizes were estimated using the Scherrer equation (eqn (2)), where *D* is the mean particle size, *K* is a dimensionless shape factor that typically accounts for the shape of the particle and is generally taken to have the value 0.94–0.97, *λ* is the X-ray wavelength, *β* is the line broadening at half of the maximum intensity (FWHM), and *θ* is the Bragg angle in radian units.2
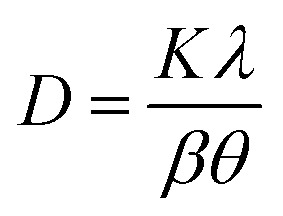


The H_2_-TPR and CO_2_-TPD experiments were carried out in calibrated ChemBET Pulsar (AntonPar) connected with a thermal conductivity detector (TCD). Typically, 50 mg of sample was loaded in a quartz U-shaped reactor, and the reducibility profile was recorded using 10% H_2_/N_2_ up to 900 °C with a heating rate of 10 °C min^−1^. For CO_2_-TPD, 50 mg of each sample was pre-reduced in 10%H_2_/N_2_ gas at 600 °C using a 10 °C min^−1^ heating rate and cooled down under the He atmosphere (30 ml min^−1^). The pre-reduced samples were exposed to 10% CO_2_/N_2_ for 45 min. Then, the physisorbed CO_2_ species were flushed out using He for 30 min. The chemisorbed CO_2_-TPD profile was recorded continuously in N_2_ flow using a heating rate of 20 °C min^−1^ up to 600 °C. Fourier Transform Infrared Transmission (FT-IR) spectra were studied using the BRUKER ALPHA PLATINUM-ATR spectrometer. For experiments, a 10 mg sieved powder sample (100–200 μm) was loaded on ATR crystal to perform testing using OPUS software in the range of 400 to 4000 cm^−1^. The surface area and pore size distribution were evaluated by N_2_-physisorption measurements using Gemini VII 2390, Micromeritics, Norcross, USA) at 77 K. Before the analysis, the samples were degassed for 2 h at 350 °C under vacuum. The surface morphology of the samples was analyzed by SEM equipped with EDS using Oxford Instrument (JSM-IT300LV, JEOL GmbH, Germany). Furthermore, the cyclability of the *in situ* pre-reduced supports and the catalysts were tested through a series of oxidative and reductive cycles using METTLER Toledo TGA/SDTA 851e Thermogravimetric Instrument at 400 °C. Typically, 50–60 mg samples weighed in 40 μl alumina crucibles were reduced in 4% (H_2_/N_2_) mixture followed by oxidation (using 76 ml per min N_2_, 4 ml per min CO_2_, 20 ml per min N_2_-as carrier) and reduction (using 76 ml per min N_2_, 4 ml per min H_2_, 20 ml per min N_2_-as carrier). The reversible redox capacities of the catalysts were estimated by weight variations (wt%) under optimized reaction conditions, which was linked to their O-exchange capabilities. The surface elemental composition of the catalyst was analyzed by X-ray photoelectron spectroscopy (XPS). In addition, coke formation during the CO_2_ methanation reaction over catalysts was analyzed using METTLER Toledo TGA/SDTA 851e Thermogravimetric Instrument. A 50 mg of spent catalyst was filled in a 40 μl alumina crucible and heated from ambient temperature to 900 °C with a ramping rate of 15 °C min^−1^ under air (20% O_2_/80% N_2_).

### 2.3 Catalytic activity

**Fig. 1:**
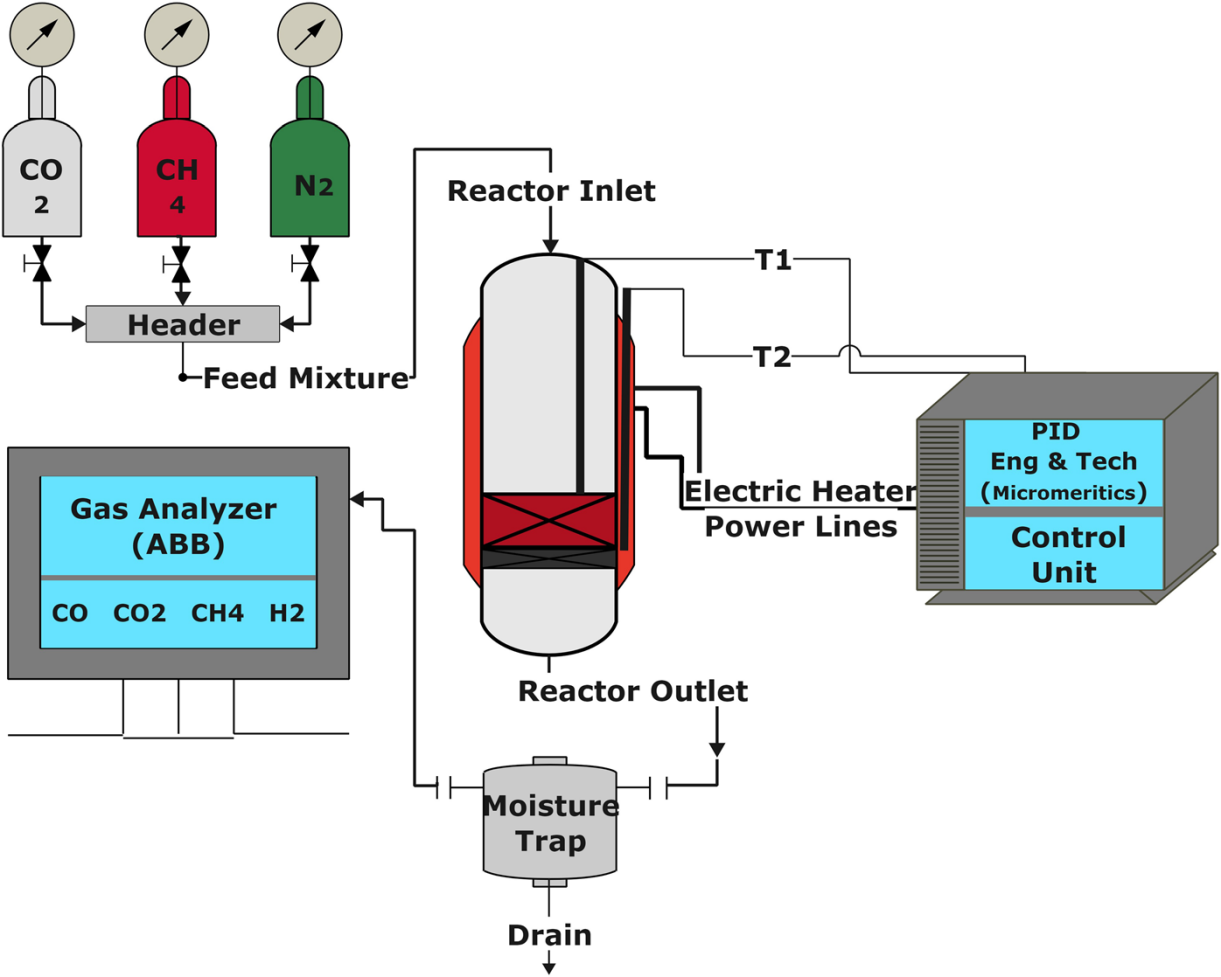
The schematic diagram for the experimental setup.

The catalytic performance of the catalysts was evaluated using a tubular reactor (inner diameter = 0.9 cm) connected with a digital interface Micromeritics PID Eng & Tech controller and ABB AO2020 analyzers, as shown in Fig. 1. The ABB analyzing set-up is equipped with Caldos 25 and Uras 26 detection housing units for continuous measurement of gas composition at the reactor outlet.

The reaction was carried out by loading 100 mg of catalyst (100–200 μm size) diluted with SiC up to 0.5 cm^3^ in the reactor with a space velocity of 12 000 h^−1^. The catalysts were *in situ* reduced at 600 °C for 30 min under a 20% H_2_/N_2_ gas mixture. Then, the reactor was cooled down to 200 °C, and the reaction was carried out between 200–600 °C at atmospheric pressure. Feed gas CO_2_/H_2_ with the ratio of 1/4 (balanced with N_2_) was introduced, and product gas composition was monitored with ABB analyzers. The exhibited catalytic activity reflects the average conversion and selectivity values over a stable temperature. Furthermore, the effect of CO_2_ partial pressure and flow rate was also investigated. The stability test was recorded at a constant temperature of 400 °C for 50 h under similar reaction conditions. The conversion and selectivities were calculated using the following (eqn (3)–(5)).3
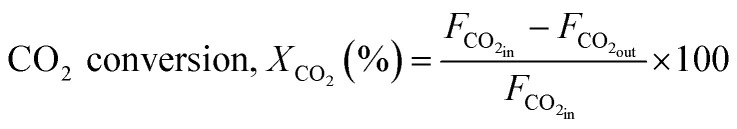
4
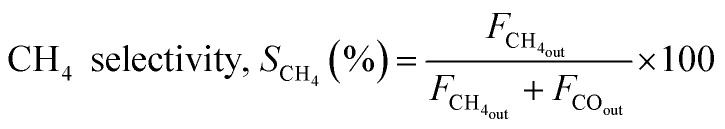
5
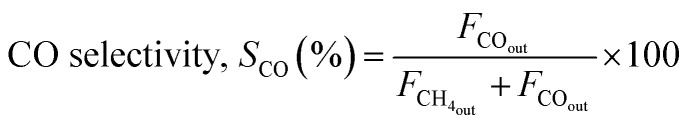


## 3. Results and discussion

### 3.1 Catalyst composition

The chemical composition of the calcined supports and catalysts determined by ICP-AES are presented in Table 1. The Ce-containing samples were analyzed using ICP-SFMS due to the limitation of the ICP-AES instrument. The elemental compositions of all the samples were close to the calculated compositions. The evaluated Ni contents were found in an acceptable range for all samples, as 10 wt% Ni was loaded into each synthesized catalyst. Assuming no impurities, the rest of the contribution in each experimental formula justifies the oxygen composition. Perovskite structure can be predicted by the GoldSchmidt tolerance factor (*t*). Ideally, the perovskite has a tolerance factor of 1 for a cubic symmetric structure. For non-symmetric structures, the tolerance factor values are in the range of 0.75 < *t* < 1, which can be calculated by the following (eqn (6)).6
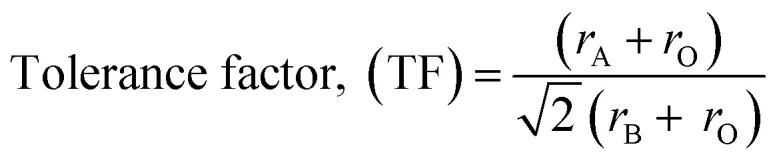
where *r*_A_, *r*_B_ and *r*_O_ represent the ionic radii of A, B, and O in the ABO_3_ perovskite structure. Table 1 lists the calculated values of the tolerance factor for perovskite supports, which are in good agreement with the non-symmetric perovskite structure.^14–17^

**Table 1:** Elemental composition of the samples (metals)

	Sm (wt%)	La (wt%)	Nd (wt%)	Ce (wt%)	Pr (wt%)	Mn (wt%)	Ni (wt%)	Experimental formula	Tolerance^a^ factor
SM	63.27	—	—	—	—	20.12	—	SmMnO_3_	0.86
NSM	52.44	—	—	—	—	13.97	8.01	Ni/SmMnO_3_	—
LM	—	62.53	—	—	—	18.21	—	LaMnO_3_	0.88
NLM	—	53.66	—	—	—	16.99	8.47	Ni/LaMnO_3_	—
NM	—	—	63.72	—	—	16.75	—	NdMnO_3_	0.87
NNM	—	—	52.44	—	—	19.05	8.02	Ni/NdMnO_3_	—
CM	—	—	—	48.3	—	21.09	—	CeMnO_3_	0.87
NCM	—	—	—	44.2	—	14.34	8.16	Ni/CeMnO_3_	—
PM	—	—	—	—	65.66	17.97	—	PrMnO_3_	0.87
NPM	—	—	—	—	53.09	12.21	8.79	Ni/PrMnO_3_	—

a From GoldSchmidt tolerance factor.

### 3.2 XRD

**Fig. 2:**
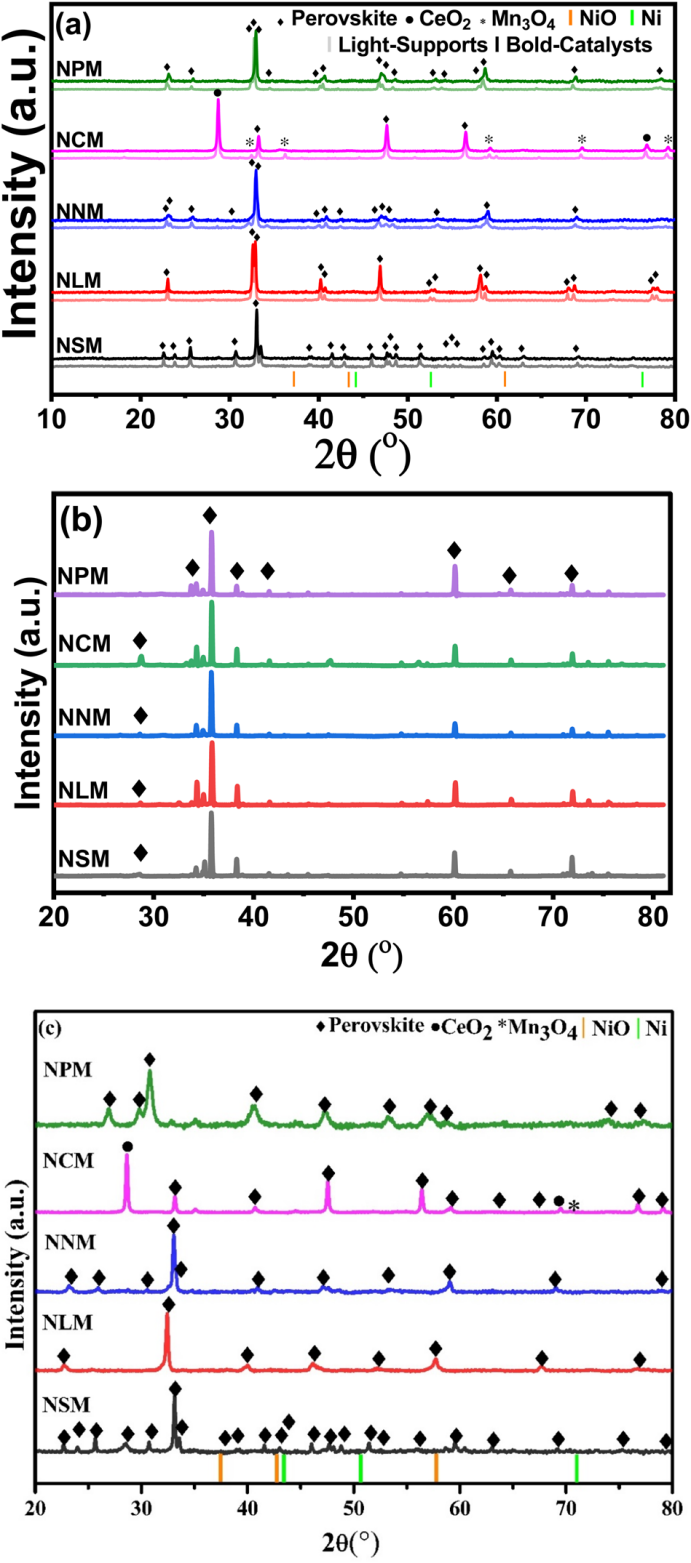
XRD diffractograms of (a) calcined supports and catalysts, (b) spent-catalysts catalysts, and (c) reduced catalysts.

XRD diffractograms displayed by the support materials (light profile) and catalysts (bold profile) are shown in Fig. 2(a). The peaks with the diamond symbol are attributed to the characteristic peaks of the perovskite crystal structure, which confirmed the successful formation of the perovskite phases. All the samples demonstrated rare-earth manganates as the main phase. Characteristics diffraction peaks of rare-earth oxides disappeared for catalysts, except CeMnO_3,_ which showed additional peaks of CeO_2_ (2*θ* = 28.65°, 76.83°) and Mn_3_O_4_ (2*θ* = 32.41°, 30.06°, 59.20°, 69.54°, 79.27°), respectively.^20–22^ It has been reported that the small amount of Ce and Mn elements might not be well incorporated into the perovskite structure.^23^ The possible reason for the presence of CeO_2_ and Mn_*x*_O_*y*_ species may be charge imbalance within the perovskite structure owing to different valence states of Ce and Mn at elevated temperatures. As a result, a minor quantity of Ce and Mn oxides appeared along with the perovskite oxide structure.

Fig. 2(b) displays the XRD diffractograms of the spent catalysts. The slight peak shift on higher 2*θ* values was attributed to higher crystallite size reflected by spent catalysts (ref. Table 2). In fact, the lattice parameters started rearranging within the structure of perovskite, which could cause a decrease in lattice parameters of spent catalysts, leading to the contraction of lattice and peak angles shifted to slightly higher 2*θ* values. It is also reflected that structural tilting brings lattice changes in the existing perovskite crystal structure that alter the lattice parameters by comprehensive strain and cause the shrinkage of the respective perovskite structure. Resultantly, the main perovskite peaks shifted at a higher 2*θ* range (2*θ* = 35.78°).^24,25^ Additionally, the XRD analysis of spent catalysts assures the structure stability of re-arranged perovskite phases. Minor peaks might disappear in the case of NLM and NPM catalysts, likely due to their characteristic instability.^18^

**Table 2:** Structural characteristics and CO_2_ and H_2_ quantification values of the synthesized materials^a^

	CS_fresh,XRD_ (nm)	CS_spent,XRD_ (nm)	CO_2,desorbed_ (μmol_CO_2__ g_cat_^−1^)	H_2,uptake_ (μmol_H_2__ g_cat_^−1^)
SM	36.27	—	—	505.10
NSM	41.32	64.50	656	723.76
LM	47.62	—	—	667.32
NLM	37.13	65.50	708	963.32
NM	18.82	—	—	714.04
NNM	22.10	50.69	575	1048.93
CM	31.17	—	—	476.80
NCM	37.25	49.60	865	1077.23
PM	29.64	—	—	601.73
NPM	26.23	50.97	740	1218.41

a CS: Crystal size estimated *via* Scherrer's equation. CO_2_ and H_2_ amounts were determined using pre-defined calibration values for the experimental concentration of both gases.

As a reference, the Ni (JCDPS 087-0712) and NiO (JCDPS 047-1049) positions are marked with vertical orange and green bars. The absence of NiO peaks in calcined catalysts accentuates the well-dispersed Ni particles.^26^ Further, the crystallite size calculated from the diffractogram of spent catalysts increased, which shows possible sintering of Ni during the reaction and has been reported in the literature as well. The diffractograms of reduced catalysts do not show diffraction peaks from reduced Ni metal (Fig. 2(c)). Thus, the disappearance of diffraction peaks at attributed positions justifies the uniform dispersion and incorporation of Ni into the lattice of perovskite.

### 3.3 Basic and redox properties

**Fig. 3:**
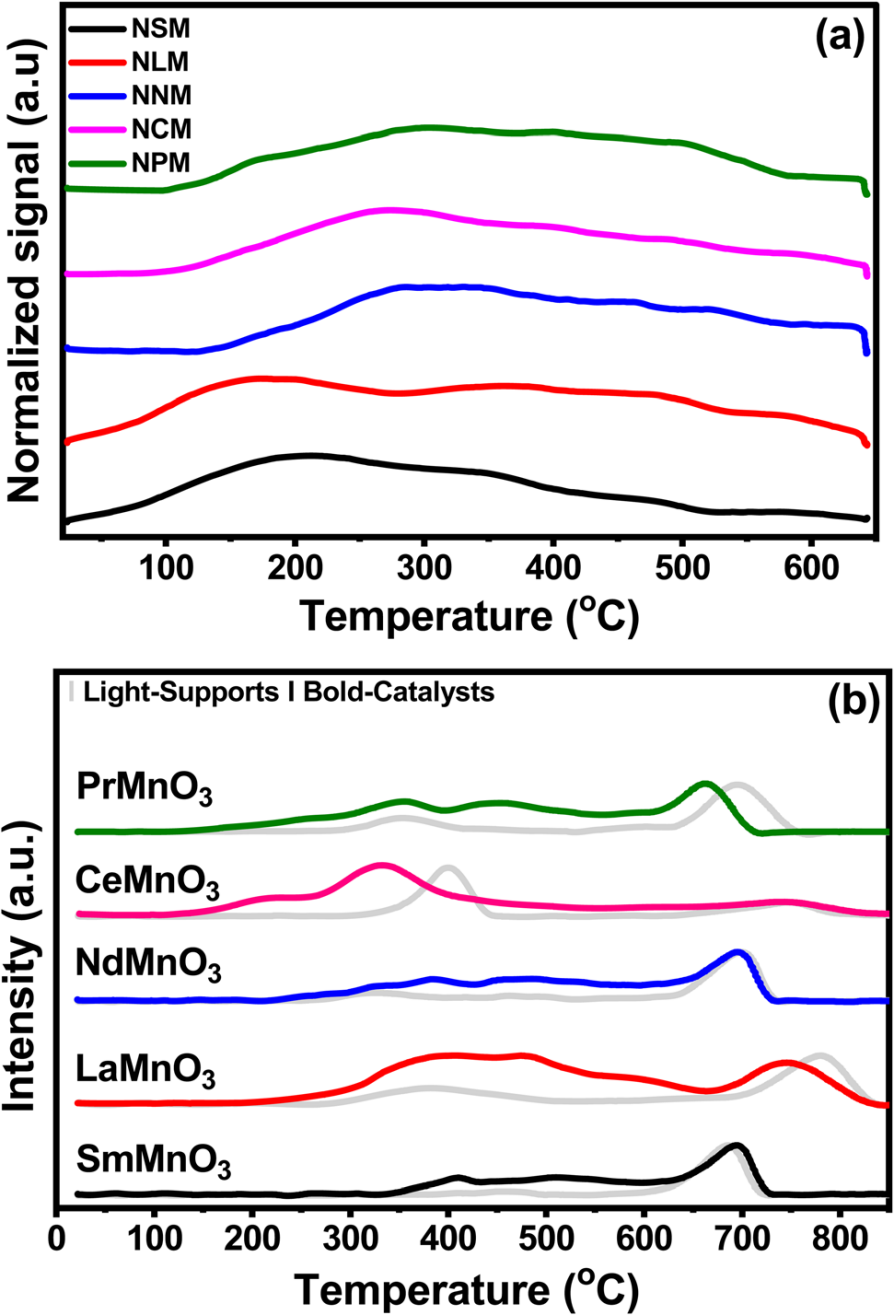
(a) CO_2_-TPD profiles of the pre-reduced catalysts and (b) H_2_-TPR of supports and catalysts.

Fig. 3(a) displayed the CO_2_-TPD profiles of the catalysts recorded as a function of temperature and the quantification of CO_2_ desorbed, which is listed in Table 2. The CO_2_ desorption profiles show weak (<300 °C), moderate (300–500 °C), and strong (>500 °C) basic sites.^27^ The CO_2_ adsorption and activation are associated with the strength of the species in the range of weak and moderate basic sites.^13^ The CO_2_ is adsorbed on the catalyst surface as monodentate over weak basic sites, whereas at medium and strong basic sites, CO_2_ is strongly attached to surface oxides in the form of bidentate.^28^ All the catalysts exhibited weak and moderate surface basic sites in a comparable range. The surface of the catalyst involved heterogeneous types of weak and strong basic sites. The CO_2_ adsorption sites with different strengths lead to inequivalent amounts of chemisorbed CO_2_ species. The reduced conditions favor the CO_2_ adsorption to cationic sites, which leads to weak adsorption of CO_2_ molecules to the catalyst surface.^29^ The NCM catalyst displayed the highest CO_2_ desorption capacity in the given temperature range, reflecting greater basic sites that would favor the CO_2_ activation mechanism. The decrease in CO_2_ desorption follows the order of NCM > NPM > NLM > NSM > NNM.

Fig. 3(b) shows the H_2_-TPR test normalized reduction profiles by the calcined support (light-pattern) paired with respective catalysts (bold-pattern). Perovskites displayed two reduction regions at 290–560 °C and 580–850 °C, respectively. The reduction peaks of Mn^4+^ to Mn^3+^ and Mn^3+^ to Mn^2+^ at lower temperature regions may be attributed to surface oxygen consumption, while the reduction peak in the higher temperature region might be associated with bulk removal of species.^30–32^ Here, the consumption of H_2_ species indicates the presence of oxygen vacancies in perovskite oxides. However, the reduction peak of Mn to Mn^0^ was not found.^33^ The Ni-doped PTOs showed strong reduction peaks for NSM, NLM, NNM, and NPM between 600–850 °C, along with two minor peaks around 290–560 °C. Minor reduction peaks below 600 °C depicted the fewer oxygen species available at the catalyst surface.

Interestingly, the NCM catalyst exhibited a notable shift in peak to a lower temperature with a side dome that underscores the presence of Ni particles, which support the earlier activation of the H_2_ to H. The low temperature reduction peaks (208 and 331 °C) are attributed to the reduction of surface oxygen species, which further contribute to forming oxygen vacancies in the Ni–PTOs interface and structure.^34^ Further, a slight shift in the reduction peak of Ni-supported LM and PM perovskites at lower temperature regions were also associated with the presence of Ni particles, promoting the lattice species reduction. In the same scenario, the NSM catalyst displayed a slightly delayed reduction, but no change was observed for the NCM catalyst at the higher temperature reduction region. Overall, the NCM catalyst showed improved redox properties with reduction peaks at a lower temperature. The quantification of the total H_2_ uptake is listed in Table 2, including the contribution of the higher redox peak at elevated temperature (>600 °C) depicted by Ni-doped SM, LM, NM, and PM except for CM, which might be attributed to the bulk reduction of O species.

### 3.4 FTIR spectra

**Fig. 4:**
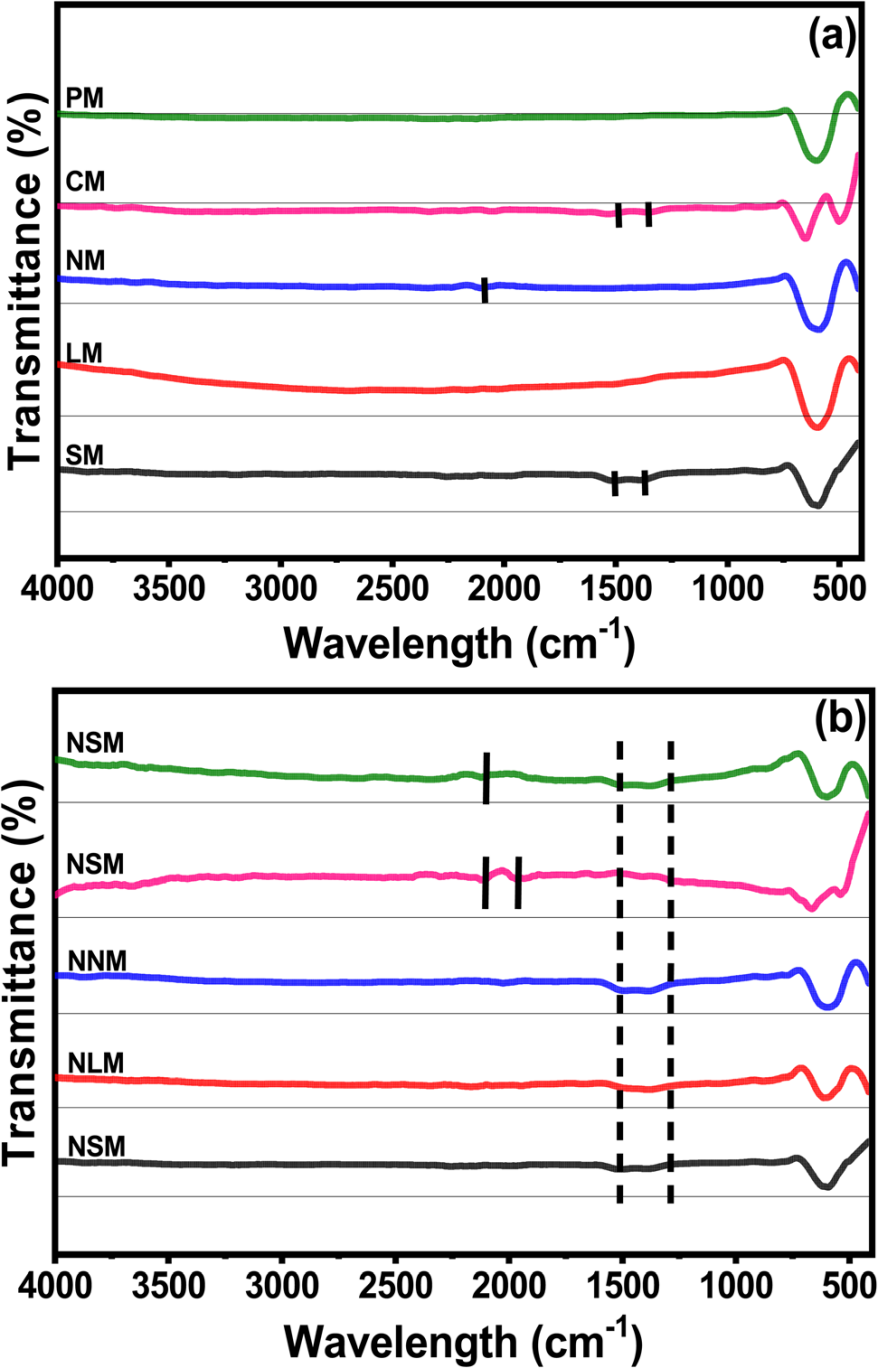
FTIR spectra of (a) supports and (b) catalysts.

Fig. 4(a and b) show the FTIR spectra of calcined perovskite supports and Ni-doped PTOs. All the samples showed strong bending characteristics around 590 cm^−1^, which corresponded to Mn–O–Mn deformation associated with the octahedron MnO_6_. The CM support displayed another bending around 488 cm^−1^ attributed to Mn–O stretching. A small vibration around 830–690 cm^−1^ indicates the presence of Sm–O, La–O, Nd–O, Ce–O, and Pr–O bonding. The marked regions between 1650–1200 and 2450–1720 cm^−1^, with tiny bands, are related to the O–H stretching of water molecules.^35–38^

### 3.5 N_2_ adsorption–desorption isotherms

**Fig. 5:**
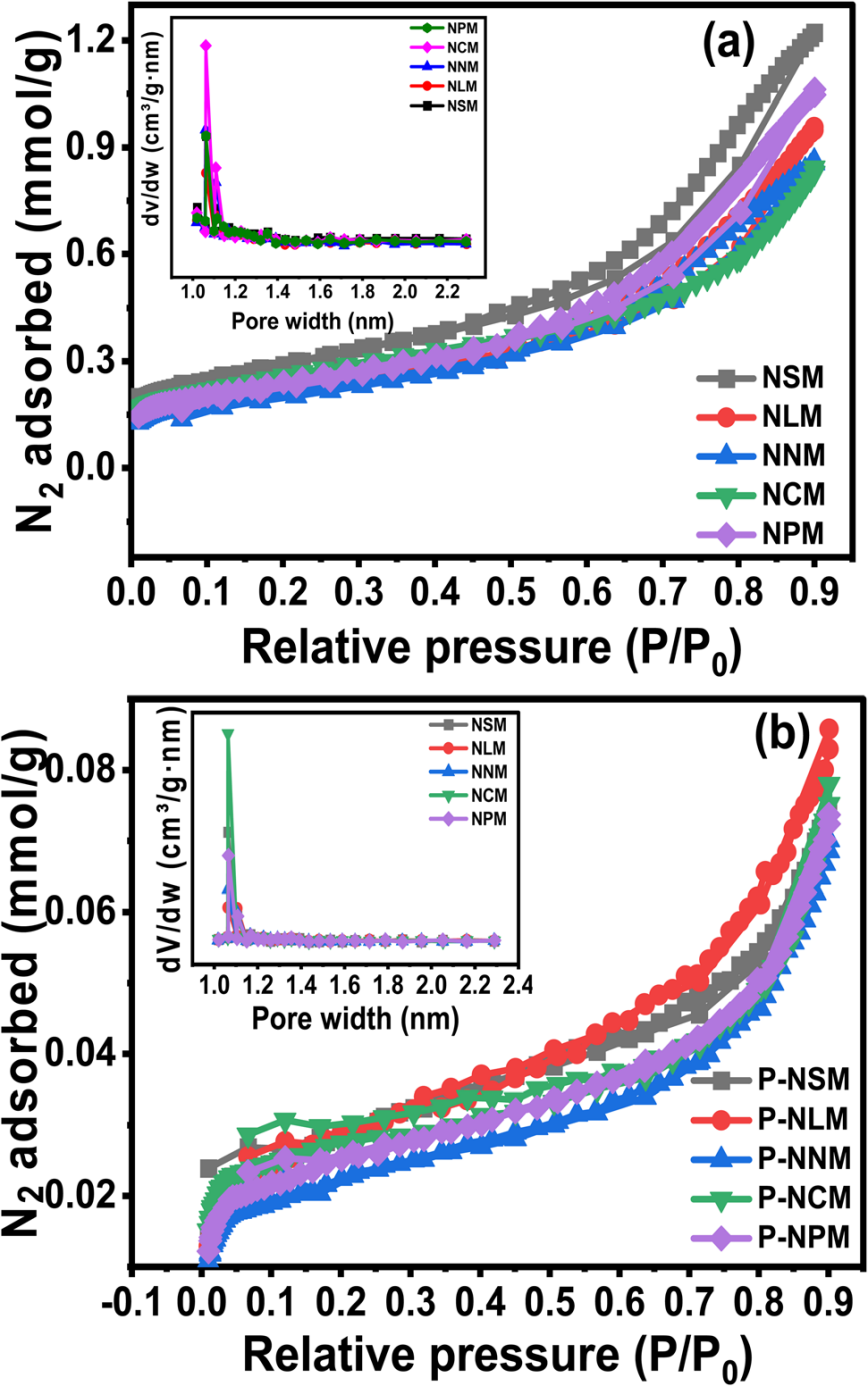
N_2_ adsorption–desorption isotherms of (a) fresh and (b) spent catalysts.

The surface properties such as surface area, pore size, and pore volume are crucially linked to the active sites, heat, and mass transfer mechanisms. The N_2_ adsorption and desorption isotherms were performed to understand the porosity and textural properties of the catalysts, as shown in Fig. 5(a and b). The isotherms showed the type IV curve with a small H3-like hysteresis as classified by the IUPAC.^39^ The inset plots showed mainly micropores (<2 nm) with a small pore distribution of mesopores (>2 nm) for synthesized and spent catalysts. The H3 hysteresis resembles slit-like pore geometry. At relatively higher pressures above (*P*/*P*_0_ > 0.4), the adsorption isotherms indicated the multiple layers deposition of N_2_ molecules. The textural properties evaluated from nitrogen sorption isotherms are listed in Table 3. The *S*_BET_ area decreases in the order: NNM < NLM < NPM < NCM < NSM. The NSM catalyst showed the largest surface area (23.1 m^2^ g^−1^), while the NNM catalyst showed the lowest surface area among the calcined catalysts. The surface area of the NCM was close to the NSM catalyst.

**Table 3:** Textural properties of the catalysts

	*S*_BET,fresh_ (m^2^ g^−1^)	*S*_BET,spent_ a (m^2^ g^−1^)	*V*_pore,fresh_ a × 10^−3^ (cm^3^ g^−1^)	*V*_pore,spent_ a × 10^−3^ (cm^3^ g^−1^)	*D*_pore,fresh_ (nm)	*D*_pore,spent_ a (nm)
NSM	23.1	2.2	6.4	0.5	1.1	0.9
NLM	17.8	2.2	4.7	0.5	1.0	0.8
NNM	17.0	1.7	4.4	0.3	1.0	0.8
NCM	21.0	2.0	5.6	0.5	1.1	1.0
NPM	19.3	1.9	5.0	0.4	1.0	0.8

a Surface area estimated for spent catalysts includes the SiC used as diluent.

According to the pore size displayed in the inset picture, NCM catalysts have larger pore sizes compared to other catalysts, which would be helpful in catalytic activity. In addition, the surface area of the spent catalysts was drastically decreased, and the trend was similar in all the catalysts. The decreased surface area could probably have resulted from the sintering of Ni particles and coke formation during the CO_2_ methanation reaction. An increase in the crystallite size of the spent catalysts was observed in the XRD diffractogram of the samples, which justifies the decrease in the surface area and pore volume of the catalysts. On the other hand, coking could have blocked the pores of the catalysts, thereby leading to reduced surface area and pore volume. Coke formation has further been analyzed from the TGA under air, as discussed later in the catalytic activity part.

### 3.6 SEM-EDS analysis

**Fig. 6:**
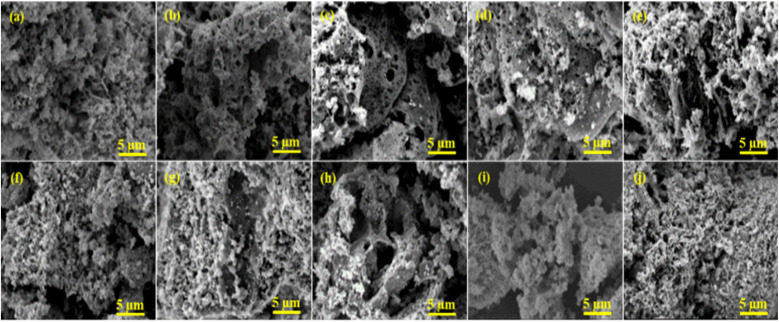
SEM images of supports (a) SM, (b) LM, (c) NM, (d) CM, (e) PM and catalysts (f) NSM, (g) NLM, (h) NNM, (i) NCM and (j) NPM.

Fig. 6 displayed the morphology and elemental composition of perovskite supports and Ni-doped PTOs investigated by the SEM-EDS. SEM micrographs revealed that perovskite supports and catalysts have a porous structure consisting of tiny, non-uniform, and irregular shape boundaries. The creation of a porous network in the solid phase for all samples linked to the solution combustion method. The evolution of gaseous products is governed by the thermal effects that tend to escape the solid surfaces, forming various pore size distributions. The porous morphology and microstructural characteristics of materials are strongly linked to gas phase catalytic activities. Both perovskite support and catalyst showed a porous structure with a certain pore width and diameter, as evaluated through N_2_ adsorption and deportation isotherms. The EDS analysis provided the elemental information of the prepared supports and catalysts, as presented in Table 4. The chemical compositions observed by EDS revealed the surface features of the catalysts. The elemental composition from EDS and ICP were quite close, with slight variations for both supports and catalysts.

**Table 4:** EDS elemental composition of the supports and catalysts

	Sm (wt%)	La (wt%)	Nd (wt%)	Ce (wt%)	Pr (wt%)	Mn (wt%)	Ni (wt%)	Oxygen (wt%)
SM	62.77	—	—	—	—	24.22	—	13.1
NSM	53.22	—	—	—	—	19.6	8.1	19.1
LM	—	65.31	—	—	—	22.54	—	12.1
NLM	—	61.4	—	—	—	20.00	8.6	10.00
NM	—	—	61.40	—	—	25.51	—	13.1
NNM	—	—	53.34	—	—	21.67	10.2	14.9
CM	—	—	—	62.9	—	24.00	—	13.1
NCM	—	—	—	55.9	—	17.6	8.5	18.01
PM	—	—	—	—	63.9	25.31	—	10.90
NPM	—	—	—	—	52.32	20.42	9.5	17.70

### 3.7 Cyclic reversibility and structural stability

**Fig. 7:**
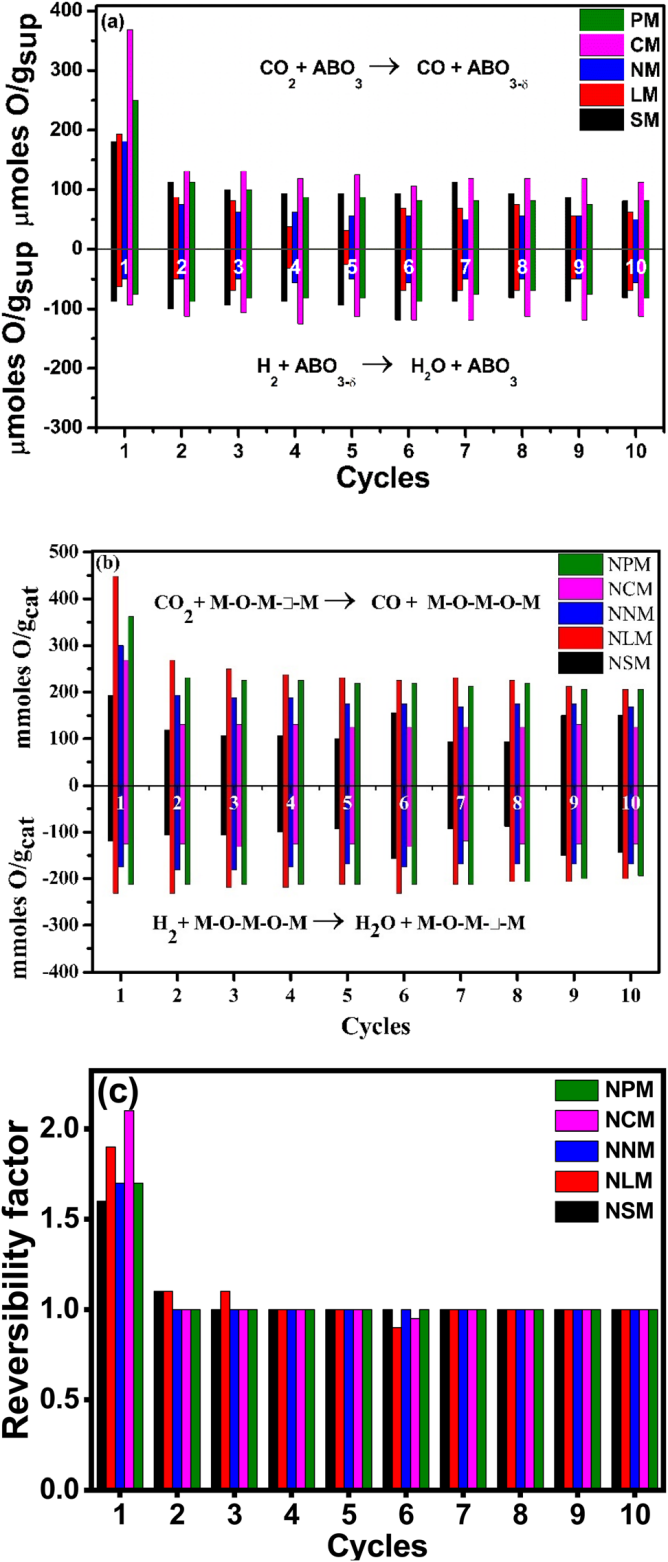
Cyclic performance of (a) supports, (b) Ni-doped catalysts, (c) reversibility of catalysts at 400 °C.

The cyclic reversibility of materials plays a crucial role in long-term application for catalytic reactions. Fig. 7(a and b) present the weight variation response for the samples when they were exposed to CO_2_ and H_2_ streams, alternatively for 10 cycles at 400 °C. The weight variation is attributed to the oxygen exchange capabilities of the materials. Except for 1^st^ cycle (where the sample was exposed after pre-reduction at 600 °C under the H_2_ atmosphere), samples showed a constant exchange capacity under redox cycles. The Ce-based magnetite perovskite displayed better oxygen exchange capacities in all redox cycles, as shown in Fig. 7(a), in comparison to different synthesized supports. Interestingly, nickel doping enhanced the oxygen exchange capacities of the La, Pr, and Nd magnate-based catalysts, whereas Sm and Ce-based Ni-doped perovskite displayed relatively constant oxygen vacancies up to 10 redox cycles. The Ni-doping promotes oxygen exchange capabilities by disturbing the charge neutrality of the lattice structure. The non-symmetric perovskite structural defects chemistry favored the oxygen mobility from surface to lattice and lattice to surface only by the oxygen vacancies^.^^40^ The cations and morphology of the samples at the A and B sites affect the oxygen capacities by regulating the valence state of Mn ions. The electronegativity principle explained the formation of Mn mixed-valence ions by excess oxygen and substituting lower-valence cations at the A or B sites.^41^

In addition, Ni doping creates the additional Mn^4+^ ions that promote oxygen capacities by adjusting the structural defects in Ni-doped LaMnO_3_. On the contrary, the disappearance of defect chemistry in perovskite structures restricts the migration of oxygen species, resulting in a reduction of lattice oxygen activities.^42^ Additionally, catalytic activity depends on available oxygen vacancies and the Ni–metal interaction, metal dispersion, reduced Ni-sites for H_2_ dissociation, the valence state of A-site element, surface area, and affinity to CO_2_ as well as H_2_O species.^43^Fig. 7(c) shows the reversibility factor of the Ni-doped perovskites corresponding to the redox cycles. It indicates that the prepared Ni-doped perovskites retained the oxygen exchange capacities in each cycle except the first cycle.^44^ The structural stability holds the key in assessing the catalyst performance, which is directly affected by harsh reaction conditions. The XRD analysis of spent catalysts displayed a stable structure indexed with pure perovskite phases, as shown in Fig. 2(b). Also, the stable perovskite structure agreed to attain the constant reversibility of Ni–PTOs over repeated cycles, as shown in Fig. 7(c). Here, the reversibility factor is estimated based on oxygen exchange capacities in each oxidative and reductive cycle for all spent catalysts. The unity value for all the samples, irrespective of 1^st^ cycle, indicated the constant oxygen diffusion related to homogenous porosity and pore size distribution over the surface of the catalyst.

### 3.8 XPS analysis

**Fig. 8:**
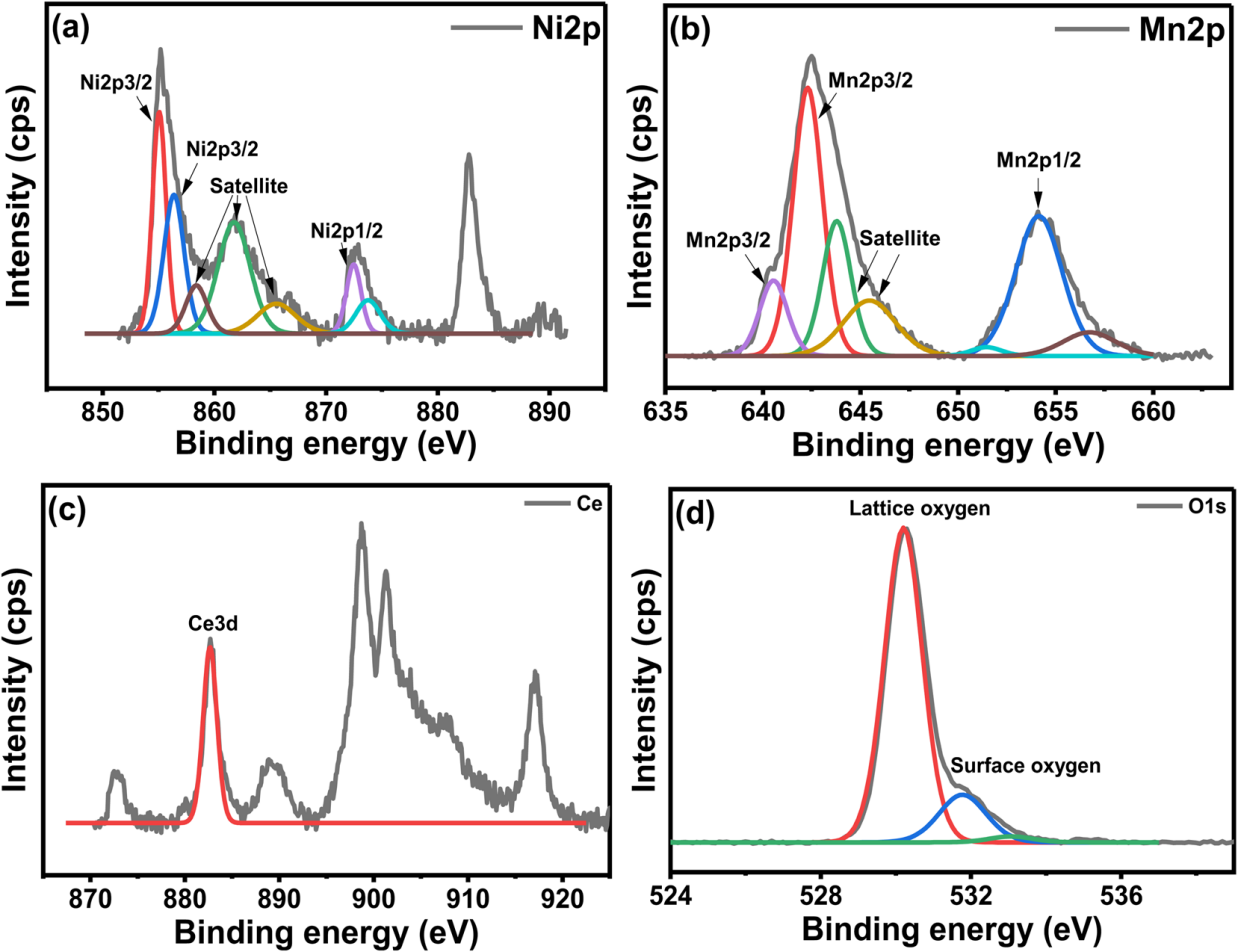
XPS analysis of the 10 wt% Ni/CeMnO_3_ catalyst: (a) Ni 2p; (b) Mn 2p; (c) Ce; and (d) O 1s.

Fig. 8 shows the XPS analysis of the NCM catalyst. The Ni deconvoluted spectra in Fig. 8(a) showed two peaks at 855.1 eV and 856.4 eV, corresponding to the NiO present on the surface and in the lattice of the perovskite, respectively.^16^Fig. 8(b) displayed Mn deconvolution spectra showed main peaks attributable to the spin orbits for Mn 2p_3/2_ and Mn 2p_1/2_ at 642.3 and 640.5 eV, revealing the prevalence of Mn^3+^ ion.^45^ These energy values represented various mixed manganese oxides, perovskite, and spinel structures, which are normally associated with Mn^4+^ and Mn^3+/^Mn^2+^ type species.^46^Fig. 8(c) indicated Ce 3d spectra, exhibiting a typical peak at 882.8 eV, corresponding to the Ce^4+^, proving an occupancy of the Ce^4+^ oxidation state in the CeMnO_3_ perovskite catalyst.^47,48^ The O 1s spectra exhibited two peaks at 530.2 eV and 531.8 eV, respectively as shown in Fig. 8(d). These oxygen peaks could be related to the lattice oxygen species of perovskite and surface-adsorbed oxygen-deficient vacancies of CeMnO_3_ perovskite, thus confirming the typical perovskite behavior. The presence of Ce in the perovskite could increase the mobility of lattice oxygen and transform it into surface oxygen, which is believed to be useful for the oxidation of carbon formed during the reaction.^49^

### 3.9 Catalytic activity test

**Fig. 9:**
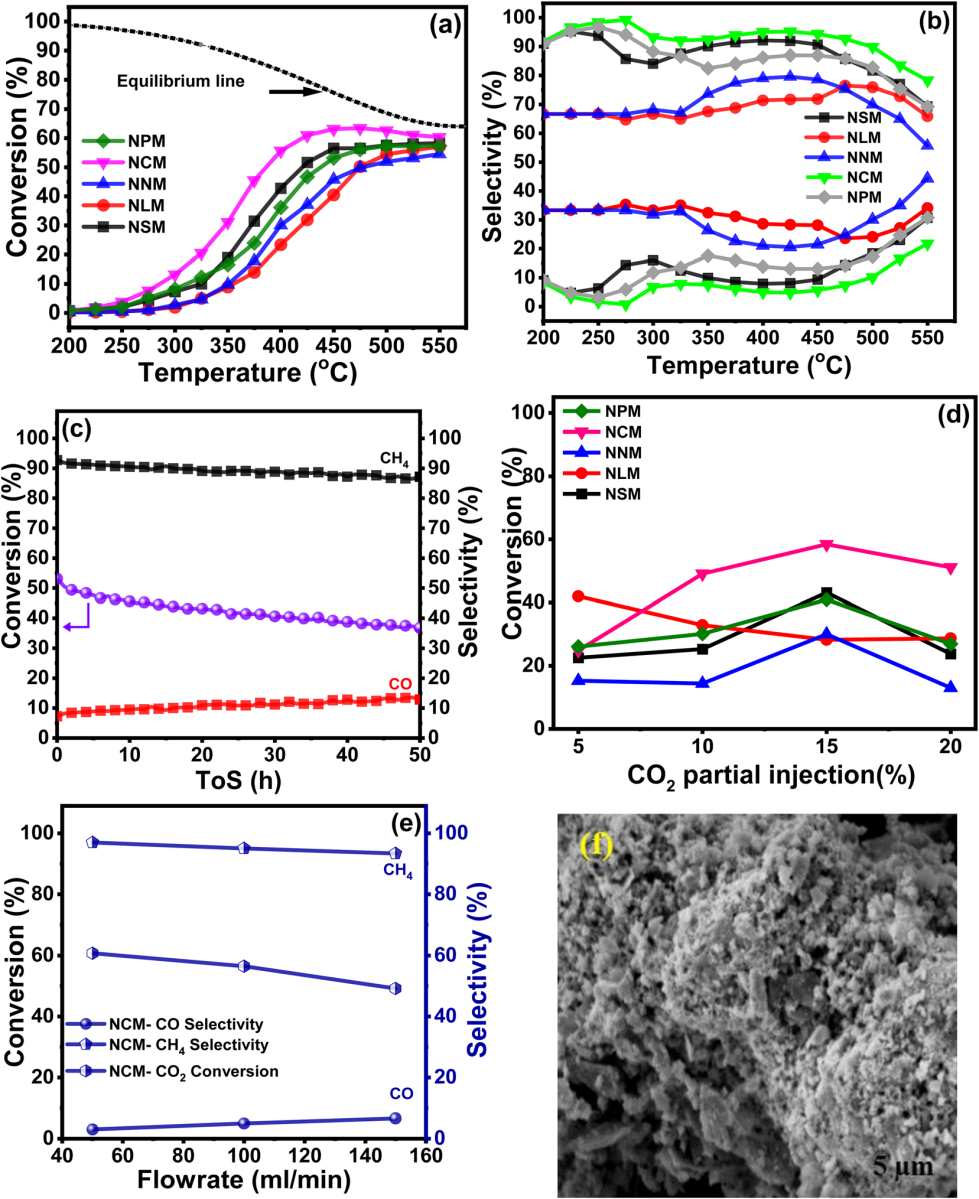
Catalytic activity at 1 atm, H_2_/CO_2_ = 4 and GHSV : 12 000 h^−1^ (a) CO_2_ conversion (%); (b) CH_4_ selectivity (%) and CO selectivity (%) (c) stability test for the NCM catalyst at 400 °C, (d) partial effect of CO_2_ concentration (e) effect of flowrate to CO_2_ conversion and (f) SEM image of spent NCM catalyst.

The catalytic activities of the Ni-doped PTOs were evaluated for CO_2_ methanation under the following reaction conditions: H_2_/CO_2_ = 4, GHSV = 12 000 h^−1^, 200–550 °C, and 1 atm. Fig. 9(a) exhibits the sinusoidal s-curve type CO_2_ conversion profiles of all the catalysts, which are kinetically controlled at lower reaction conditions and thermodynamically limited at higher temperature ranges. The exothermic nature of the CO_2_ methanation limits the reaction at elevated temperatures, which adversely drops CO_2_ conversion and CH_4_ selectivity.

Among all, the NCM catalyst achieved the highest CO_2_ conversion *ca.* 56%, with a reaction rate of *ca.* 6.6 × 10^−5^ mol_CO_2__ g_cat_^−1^ s^−1^ at 400 °C, whereas the NLM catalyst showed the lowest catalytic activity with CO_2_ conversion *ca.* 23% with a rate of 2.75 × 10^−5^ mol_CO_2__ g_cat_^−1^ s^−1^ at 400 °C. Performance analysis of the catalysts follows descending trends: NCM > NSM > NNM > NPM > NLM. Superior basicity and redox properties showed by the NCM catalyst were instrumental in activating CO_2_ molecules and resultantly enhancing catalytic activity. The dominant catalytic activity of the Ce-based oxides among the presented lanthanide metals has been reported, and results were attributed to the crucial factors that support the higher CO_2_ conversion and CH_4_ selectivity.^43^Fig. 9(b) displayed % CH_4_ and % CO selectivities over Ni-based PTOs at different temperatures. Interestingly, catalysts revealed varying selectivity profile data, where NCM catalyst showed the highest CH_4_ selectivity than CO. The CO selectivity slightly increased at higher temperatures. It shows that an increase in temperature gradually decreased the CH_4_ selectivity. A possible reason may be triggering the reverse water–gas shift reaction (RWGS) with the rise in temperature, which supports CO production, thereby decreasing the CH_4_ selectivity.^50,51^

Among Ni-based perovskite catalysts, NCM catalyst depicted *ca.* 2.4 times higher CO_2_ conversion (%) values than NLM catalyst at 400 °C. The better conversion performance is crucially attributed to multiple supporting factors that promote the reaction mechanism. The ongoing catalytic process on the catalyst surface is positively associated with surface area, and heat and mass transfer distribution inside the catalyst is strongly influenced by the pore size characteristics of the catalysts. The homogeneous and uniform pore size mainly contributes to the CO_2_ conversion, providing a chance to carry a chemical reaction with better conversion rates at the outer surface but also inside the pores of the catalysts. The BET analysis linked with Fig. 5, and SEM micrographs (Fig. 6) revealed that higher performance of the NCM catalyst attributed to the better surface area, uniformly distributed microchannel, and homogenous orientation of the Ni-doped PTOs structure. The structural stability also served a pivotal role in the sustainable conversion rate. Also, the XRD studies of the spent-catalysts samples showed Ni–perovskite structural stability (Fig. 2(b)).

Fig. 9(c) shows the stability test for the NCM catalyst carried out at 400 °C with high CH_4_ selectivity. The catalyst demonstrated a slight decline in conversion and CH_4_ selectivity during 50 h. The gradual drop in CO_2_ conversion is associated with specific agglomeration and deactivation due to the coking phenomena of Ni-based catalysts. The SEM micrograph of the spent NCM reflected the sign of agglomeration and distorted structure, as shown in Fig. 9(f), which reduced the available surface for reactants and subsequently decreased the CO_2_ conversion. There was also increased crystallite size in the XRD pattern of spent catalysts, which shows the sintering was happening and might have led to a reduction in the CO_2_ conversion efficiency of the catalysts. The sintering of Ni-based catalysts is not unusual and has been a constant challenge in the development of stable catalysts. Sintering leads to aggregation of Ni nanoparticles and reduces the active surface area of the catalysts, thus leading to decreased catalytic activity. The surface and pore volumes of the spent catalysts were substantially lower than the synthesized catalyst, which reaffirms the sintering and coke formation. The coke formation can lead to blockage of the pores and also wrapping of the Ni nanoparticles, rendering them inaccessible to the reacting gases. The reduction in the pore volume shows the coke deposition. Although Ce-based catalysts are good at consuming the coke and show better resilience, but decrease in activity cannot be fully avoided because both the sintering and coking are happening with time and can multiply the negative impact on the mitigating catalytic performance. Furthermore, the effect of the CO_2_ partial pressure was evaluated while keeping the other reaction parameters constant, as shown in Fig. 9(d). The highest CO_2_ conversion was exhibited by the NCM catalyst at 15% CO_2_ injection at a ratio of H_2_/CO_2_ = 4 (balanced with N_2_). The CO_2_ valorization is limited due to the chemical inertness of carbon dioxide molecules. Studies presented that CO_2_ concentration is associated thermodynamically with an optimal range window to achieve the equilibrium CO_2_ conversion. The strongly inert nature of the C=O double bond hindered the reaction mechanism at lower and higher CO_2_ partial concentrations away from moderate CO_2_ limits.^52^ Moreover, for the catalyst series, experiments were also conducted to study the effect of different feed flow rates on % CO_2_ conversion at 400 °C, as shown in Fig. 9(e). It revealed that comparatively, the lower feed flow rate favored the better CO_2_ conversion.

**Fig. 10:**
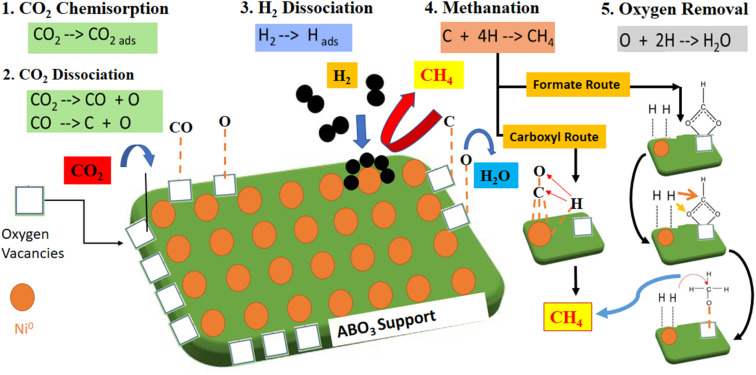
Schematic mechanism of CO_2_ methanation showing the various possible pathways.

Table 5 compares catalytic activity for recently published Ni perovskites catalysts regarding CO_2_ methanation reaction. Different preparation strategies and metal loading and reaction conditions were investigated. Compared to the listed Ni-based perovskites, the NCM catalyst prepared by the solution combustion method in this work exhibited competitive performance, attaining a remarkable CO_2_ conversion into methane. In CO_2_ methanation, the redox reaction system improved with proper A/B site cationic substitutions with variable valence metals, *i.e.* (Ce with Mn and Cu as varying valence metals) in the perovskite oxide structure. Here, the A-site cations Ce^3+^/Ce^4+^ with variable valence metal (or Mn^2+,3+,4+,^*etc.*) reversibly shuttled between PTOs and cationic oxides, which favors the perovskite support nature and resultantly improved the catalytic activity.^53^ The synthesis approach and the cationic substitutions helped to tune the physiochemical properties and promote the catalytic activity. In fact, the catalyst fabrication methods envisaged to attain better characterization results such as hydrogen consumption, CO_2_ uptake capacity, high surface area, and uniform dispersion. These factors support the promoted catalytic performance while activating the reaction molecules, favoring the CO_2_ hydrogenation reaction through associative or dissociate mechanisms, as shown in Fig. 10.^54,55^ Catalytic CO_2_ methanation can proceed through two reaction pathways, such as associative and dissociative mechanisms. In the dissociative mechanism, the CO_2_ molecules chemisorb and dissociate, constituting the carbonyl intermediates. For the associative mechanism, CO_2_ is molecularly chemisorbed, and the oxygen of CO_2_ reacts with hydrogen in sequential steps. Nevertheless, the dominant reaction pathway depends on the catalyst's nature, structure, and operating conditions.^50^ For the redox-type perovskite materials, higher reaction rates are related to (i) bifunctional surface where CO_2_ molecules are activated over available O-vacancies provided by support and adsorbed H_2_ dissociates over active Ni metal and (ii) the generation of monodentate carbonate intermediates, characterized by faster decomposition rates and associated to improved rates for methane formation.^6^

**Table 5:** Comparison of the performance of Ni-based perovskite catalysts

Catalyst (% Ni content)	Temperature (°C)	H_2_ : CO_2_ ratio	CO_2_ conv. (%)	Space velocity (L g^−1^ h^−1^)	Ref.
NCM (10%)	425	4	61	60	This work
NYMAl5 (10%)	400	4	64	60	6
Ni@CTO (10%)	350	4	80	48	15
NYM (10%)	400	4	61	60	16
La_0.6_Ca_0.4_NiO_3_ (27.3%)	300	4	59	0.05	56

Within the catalyst series, the superior catalytic performance displayed by the Ni–CeMnO_3_ could be related to the promoted generation and H-assisted decomposition of reaction intermediates, resulting in efficient metal–support synergy. Low-temperature activity of Ni/CeMnO_3_ towards CO_2_ methanation could proceed through a formate route mechanism.^57^ Both the oxide support and metallic Ni sites can interplay in the CO_2_ hydrogenation process. For the Ce-containing catalysts, bidentate carbonate species originated the formate intermediates, which are adsorbed on the oxygen vacancies. The Ce ions in the lattice structure are in intimate contact with the active metal Ni^0^, available at the metal–support interface. Therefore, CO_2_ adsorption over redox supports having Ce ions in the catalysts facilitating the formation of bidentate carbonate species and stabilizing the formate intermediates over the surface. The Ce ions concentration within redox support escalates the transformation of formate intermediates into methane formation over O-vacancies. Therefore, CO_2_ hydrogenation over Ni–CeMnO_3_ could follow the associative mechanism for CO_2_ methanation by formate route. Thus, the better oxide surfaces, smaller crystal sizes, and distorted structures attained in the Ni–CeMnO_3_ sample, reflected in the promoted redox behavior, should encourage the activation of CO_2_ molecules and dispersed Ni particles favor the hydrogenation of reaction intermediates *via* H_2_-spillover effects.

### 3.10 TGA analysis of spent catalysts

**Fig. 11:**
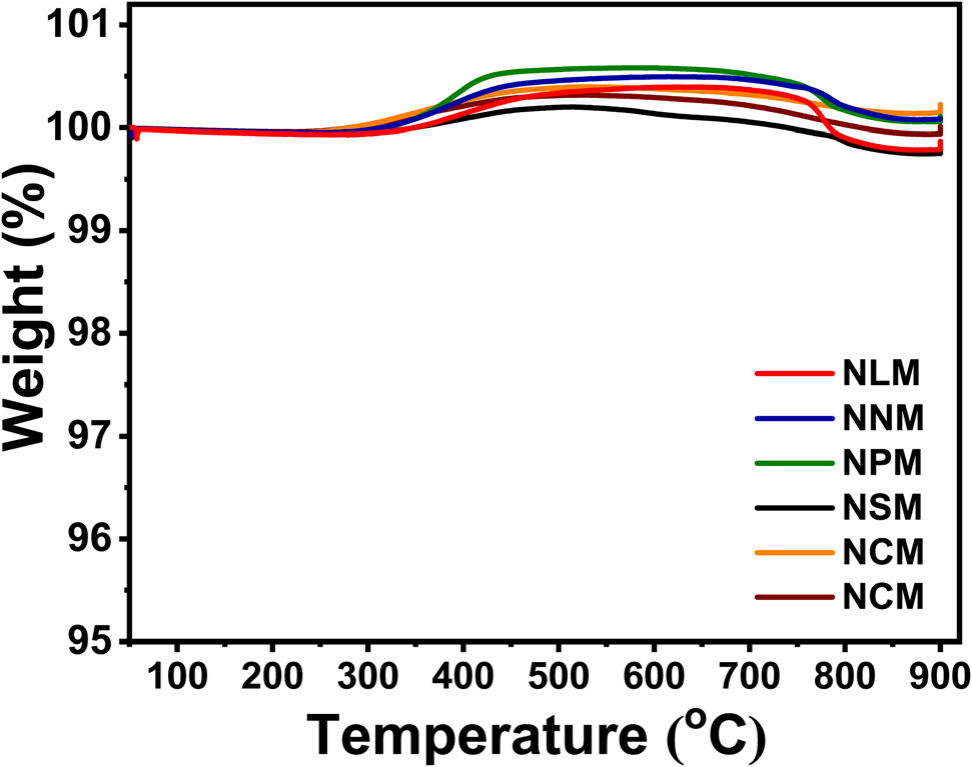
TGA analysis of the spent catalysts.

Fig. 11 shows the TGA profiles of the spent catalysts under a gas mixture of 20% O_2_ balanced with 80% nitrogen to estimate the carbon deposition on the catalysts. In all the spent catalysts, no sign of reverse oxidation of each sample was observed below 400 °C. Although, the spent Ni/perovskite catalysts exhibited the typical weight increase phenomenon in the range of 253 °C to 796 °C, which corresponds with the oxidation of metallic Ni on the surface of catalysts.^58^ Subsequently, all curves of spent catalysts depicted a slight weight loss at an elevated temperature of 800 °C to 900 °C, which might be attributed to carbon oxidation. The obtained results showed that the spent NCM-activity sample has less decline in weight loss compared to the rest of the used catalysts. However, there was no significant weight loss behavior to explore the carbon deposition amount for these best-performing samples for CO_2_ methanation activity along with the used long-term NCM-stability catalyst. Although difficult to quantify, the decrease in mass loss at high temperatures shows that there was slight coke formation. This might have contributed toward the reduced pore volume and surface area of the catalysts, thus leading to decreased catalytic performance with time.

## 4. Conclusions

In this study, Ni-based (AMnO_3_, A = Sm, La, Nd, Ce, Pr) perovskites catalysts were synthesized *via* the auto-combustion method, characterized, and evaluated for CO_2_ methanation. The Ni/CeMnO_3_ (NCM) catalyst exhibited a higher CO_2_ conversion of *ca.* 56% at 400 °C and GHSV of 12 000 h^−1^. The incorporation of Ni species into the lattice provided additional surface area within the porous structure and promoted the reaction mechanism with higher active sites, as reflected by the performance of the NCM catalyst. On the other hand, Ni-based La perovskite catalyst exhibited the lowest activity (*ca.* 23% conversion) due to strongly chemisorbed species on the surface, resulting in fewer active sites for reaction. The NCM catalyst demonstrated remarkable resistance to coking and exhibited the least amount of crystallite aggregation, as evidenced by the analysis of the spent catalyst. Moreover, Ce could promote the redox process at the metal–support interface. Thus, highly reducible perovskite support established the adsorption and activation mechanism, and the extent of metal–metal synergy significantly improved the catalytic activity and suppressed the coke formation during CO_2_ methanation. The encouraging CO_2_ methanation results displayed by the Ni-based CM–perovskite catalyst highlighted an innovative prospect toward the adaptability of perovskite-supported catalysts in process engineering applications.

## Author contributions

Muddasar Safdar: conceptualization, investigation, methodology, formal analysis, data curation, writing-original draft; Nasir Shezad: investigation, visualization, formal analysis, data curation, writing – review & editing; Farid Akhtar: supervision, writing – review & editing; Harvey Arellano-García: supervision, writing – review & editing, project management, funding acquisition.

## Conflicts of interest

The authors declare that they have no known competing financial interests or personal relationships that could have appeared to influence the work reported in this paper.
